# Towards Goal-Directed Navigation Through Combining Learning Based Global and Local Planners

**DOI:** 10.3390/s19010176

**Published:** 2019-01-05

**Authors:** Xiaomao Zhou, Yanbin Gao, Lianwu Guan

**Affiliations:** College of Automation, Harbin Engineering University, Harbin 150001, China; gaoyanbin@hrbeu.edu.cn (Y.G.); guanlianwu@hrbeu.edu.cn (L.G.)

**Keywords:** robot navigation, global planning, end-to-end learning, local planning, reinforcement learning

## Abstract

Robot navigation is a fundamental problem in robotics and various approaches have been developed to cope with this problem. Despite the great success of previous approaches, learning-based methods are receiving growing interest in the research community. They have shown great efficiency in solving navigation tasks and offer considerable promise to build intelligent navigation systems. This paper presents a goal-directed robot navigation system that integrates global planning based on goal-directed end-to-end learning and local planning based on reinforcement learning (RL). The proposed system aims to navigate the robot to desired goal positions while also being adaptive to changes in the environment. The global planner is trained to imitate an expert’s navigation between different positions by goal-directed end-to-end learning, where both the goal representations and local observations are incorporated to generate actions. However, it is trained in a supervised fashion and is weak in dealing with changes in the environment. To solve this problem, a local planner based on deep reinforcement learning (DRL) is designed. The local planner is first implemented in a simulator and then transferred to the real world. It works complementarily to deal with situations that have not been met during training the global planner and is able to generalize over different situations. The experimental results on a robot platform demonstrate the effectiveness of the proposed navigation system.

## 1. Introduction

Robot navigation techniques are ubiquitous in domestic tasks since the ability to navigate efficiently within an environment is the prerequisite to complete many motion-related tasks. Robot navigation tasks can be simply concluded as to endow a robot with the ability to move from its current position to a designated goal location based on the sensory inputs from its onboard sensors. Conventional approaches [1,2,3,4] usually solve this problem by dividing it into several different phases including map building, localization, obstacle detection, and path planning. Although decomposing navigation in this manner allows each stage to be developed independently, it prevents each from exploiting the specific needs of the other. For example, classical SLAM based approaches [5,6] could build a metric-precise occupancy map of the environment but planning based on such a cumbersome world representation can become inconvenient and lacks flexibility due to the loss of semantic information. Also, when using a multi-step approach, each step in the process can generate errors that will be accumulated through the whole process, which affects the performance of the later steps. For example, the performance of the action planning in SLAM depends on the accuracy of the built map which depends on the performance of the perception module in the earlier step.

Recently, approaches based on deep learning have offered a way to tightly couple different phases, achieving impressive results on a large set of tasks [7,8,9]. In terms of robot navigation, without going through explicit world models or state estimation steps, learning-based approaches go directly from egocentric observations to actions, aiming at learning behaviors from data or experiences. Relevant methodologies can be divided into two main categories: Methods based on supervised learning [10] and methods based on reinforcement learning (RL) [11]. Each sub-field has been well studied and their advantages and disadvantages have been discussed a lot [12,13]. For supervised learning, it shows great efficiency in dealing with problems with large-scale data but shows a poor generalization to cases which deviate too much from the training ones. For RL, its main drawback is its low learning efficiency, especially in solving large-scale problems with sparse and delayed rewards. This makes it a challenging problem to use reinforcement learning to train a robot to navigate in a large environment. However, RL offers a powerful tool to train generalizable agents.

In this work, we focus on solving the task of enabling a robot to perform goal-directed navigation in a dynamic environment using learning-based approaches, where both learning approaches mentioned above are used. Specifically, we propose a goal-directed end-to-end learning approach to train the robot to imitate an expert’s navigation between different positions in the environment and use RL to train the robot to deal with the potential changes in the environment. End-to-end learning (imitation learning) is a supervised learning approach and is receiving lots of interest as a promising direction to train autonomous navigation systems. It imitates the expert behaviors in the training data by constructing a direct mapping from local observations to actions, for example, mapping camera images to steering and acceleration commands [14]. Although it has been successfully applied in applications like lane following [15] and off-road obstacle avoidance [16], it has never been scaled up to more complex tasks like goal-directed navigation. An important reason is that standard end-to-end learning implicitly assumes that the optimal action can be inferred from the perceptual input alone, which often does not hold in practice. For example, upon reaching an intersection, the robot’s decision to turn left, right or go straight is not just dependent on its local-view scene, but also depends on the goal location. With this in mind, we extend the end-to-end learning to solve the goal-directed navigation by incorporating the goal as a part of the perceptual input. For the goal generation, we take advantage of the semantic structure behind an environment and enable the robot to perform goal-directed navigation on top of a high-abstract semantic map. In this navigation task, a semantic representation of the environment is used to provide the high-level goal, such as the kitchen and the corridor, for the robot to navigate to. The action policy trained by the goal-directed end-to-end learning depends on the structure of a particular environment and enables the robot to navigate to the goal on the global scale by making correct turning at certain intersections based on the goal position. We call it the global planner. However, it inherits the limitation from supervised learning and tends to work well only in a relatively static environment which is similar to the one where the training happened. Considering that a robot’s working environments are not guaranteed to be fixed, especially for indoor environments which are inhabited by humans. For example, objects placed randomly on the floor temporally change the local structure, but the global structure of the environment stays. In these scenarios, the action policy trained by the end-to-end learning probably will not work well in these particular areas. To solve this, we build a second planner that works temporarily and is specialized in dealing with these situations, i.e., avoiding objects in the local areas. In particular, considering the diversity of these situations, for example, objects of different sizes and shapes can be placed at different positions, it should be able to generalize over many different situations. This planner is trained based on RL and works on the local scale. It is called the local planner. By combining the goal-directed end-to-end learning-based global planning and reinforcement learning-based local planning, the proposed navigation system can navigate the robot robustly to the goal position in an environment with potential changes in certain regions.

The proposed navigation system leverages the efficiency of using supervised learning in solving large-scale problems and the advantage of using RL in training generalizable agents. In terms of training the global planner to be environment-specific and the local planner to be generalizable, it conforms to the fact shown in humans’ navigation. For example, in order to reach the goal in an environment, we need the global information of that particular environment to plan our route, but we only need the information in the local area when doing collision-free movement and this is irrelevant to the global structure of the environment. In particular, this paper presents the following contributions:

- We present a goal-directed navigation system that integrates the global planning and the local planning which are both based on learning-based approaches
- We extend the standard end-to-end learning to the goal-directed end-to-end learning algorithm that incorporates both the local observation and the goal representation in predicting actions. The proposed algorithm is used to train a global planner that predict actions conditional on the goal in the goal-directed navigation.
- We use deep reinforcement learning to train a local planner that performs object avoidance behaviors. Dueling architecture based deep double Q network (D3QN) is adopted. The planner is trained in the simulation and then transferred into the real world.
- Extensive experiments in both the simulation and the real world are conducted to demonstrate the efficiency of the proposed navigation system.

For the following parts of the paper: Section 2 gives a brief review of the learning based approaches for robot navigation and the concept of combining the global and local plannings for navigation. Section 3 introduces each component of the proposed navigation system including the proposed goal-directed end-to-end learning, D3QN, and corresponding implementations. Section 4 presents the experiments, results, and comparisons, and demonstrates the efficiency of the proposed navigation system in solving goal-directed navigation tasks. Finally, Section 5 concludes the paper and points out the future work.

## 2. Related Work

Conventional navigation systems [5,6] rely heavily on detailed and highly accurate world representations, e.g., a geometrically precise metric map, which is computationally expensive and lacks semantics. Benefiting from recent advances in deep learning, deep learning related methods have been considered as a promising approach to address many robot navigation tasks more efficiently, aiming at directly learning control policies from raw sensory data using deep neural networks.

### 2.1. Robot Navigation Based on Supervised Learning

Supervised learning methods provide a straightforward way to train autonomous robot navigation by learning from data. An intuitive idea is to map sensory inputs directly to action predictions. For example, ALVINN [17] presented an early example of mapping images to action predictions with a shallow network for autonomous car driving. NVIDIA [15] followed the same idea by using modern deep convolution networks. Pfeiffer et al. [18] mapped the laser range findings to the moving commands. There are also many different learning models in the literature and related learning including: Drivable routes [19], near-to-far obstacle detectors [20], reactive controllers [21], and driving affordance [14]. On the other hand, imitation learning (end-to-end learning) can also be regarded as a supervised learning approach, where an agent learns to imitate the expert behaviors by observing the demonstrations performed by some experts. Due to its simplicity and efficiency, end-to-end learning has been applied in many tasks, such as autonomous flight [22], robot grasping [23], road following [24], and off-road obstacle avoidance [16]. However, since it is based on the assumption that the optimal action can be inferred from the perceptual input alone, which does not hold in many situations, it has never been applied to solve more complex tasks like goal-directed navigation. Recent works have shown the importance of incorporating the goal in solving navigation tasks. For instance, Tai et al. [25] trained a mapless motion planner to navigate a robot to desired targets, where the target position in the robot’s coordinate framework is explicitly used as a part of the input. Zhu et al. [26] showed that the action policy trained based on both the current state and the goal could have great efficiency in navigating towards the target and good generalizability. Moreover, Codevilla et al. [27] proposed a conditional imitation learning approach to train a robot to be able to make responses to high-level navigational commands. Inspired by these works, we propose a goal-directed end-to-end learning method to train an action policy that is conditional on both the current state and the goal.

### 2.2. Robot Navigation Based on Reinforcement Learning

There have been a number of recent works that use deep reinforcement learning (DRL) methods to solve robotic tasks. This is popularized by the seminal work [11] which utilized deep neural networks for the function estimation of value-based RL. Since then, a large body of variations has been seen in the literature, e.g., adaptations to continuous control [28], improvements on stabilizing its performance [29,30], and learning efficiency [31]. In terms of RL for robot navigation, Xie et al. [32] proposed an RL-based method to avoid obstacles using a monocular camera. Tai et al. [25] trained a mapless motion planner that generates continuous action commands, which was trained in simulation and could be directly applied to unseen virtual and real environments. Zhu et al. [26] addressed the task of navigating towards a visually specified target based on the images at the current and the target positions. Since RL requires an agent to interact with the environment through a trial-and-error process, where the robot may suffer irreparable damages during learning, most training is implemented in a virtual environment. Previous works have shown that the learned knowledge from the simulation can be transferred to the real world successfully [25,26].

A major challenge in RL is learning in environments with delayed or sparse rewards. To address this, Chentanez et al. [33] proposed an intrinsically motivated learning algorithm where agents are constantly encouraged to explore new areas in order not to over-explore familiar areas, which can greatly increase the learning efficiency. Mirowski et al. [34] introduced auxiliary tasks that let the agent solve some intermediate tasks while simultaneously solving the main task, where the auxiliary tasks can help the agent learn better representations that improve the performance on the main task. For learning complicated navigational behaviors with long-range planning, Kulkarni et al. [35] proposed a hierarchical RL architecture that learns over different levels of temporal abstractions, where a complicated behavior can be decomposed into several simpler ones that are more easily learned. In this work, we utilize the RL to train a local planner to perform obstacle avoidance behaviors. The task is limited to a local scale where the state and action spaces are relatively small, thus does not suffer from these problems. Moreover, training generalizable agents is always desired in RL, where agents trained for one task in a specific environment should also perform well in new environments. For this aim, a popular solution is the domain randomization technique [36,37,38] by learning from a diverse set of environments. Following this idea, we let the robot to learn from different situations in the simulation in order for better generalizations.

### 2.3. Combing Global and Local Planners for Robot Navigation

The idea of combining global and local planning has been adopted by many robot navigation systems [39,40,41]. Among them, the ROS navigation package [42] is a standard toolbox for SLAM and path planning. Typically, global and local planners work in different ways. The global planner takes the robot’s current position and the goal and calculates the trajectory with respect to a global map. The local planner is able to perceive the local information with a higher accuracy, for example, detecting more obstacles. It is responsible for amending or optimizing the trajectory proposed by the global map. In the literature, there exist many path planning methods [43]. For instance, Brock et al. [44] proposed a global dynamic window approach that combines path planning and real-time obstacle avoidance, allowing robots to perform high-velocity, goal-directed, reactive motion in unknown and dynamic environments. Ferrer et al. [45] introduced an anticipative kinodynamic planning (AKP) approach for robot navigation in urban environments, where a human motion prediction algorithm was seamlessly integrated into the planning algorithm. The resulting planning trajectory was able to minimize the impact of the robot to nearby pedestrians. Similarly, Mehta et al. [46] adopt Multi-Policy Decision Making (MPDM) to realize autonomous navigation in dynamic social environments. However, in their work, the robot’s trajectory was selected from a set of closed-loop behaviors whose utility can be predicted rather than being explicitly planned.

For learning based approaches, Wei et al. [47] introduced a two-level navigation model that integrates model-free deep learning with model-based planning where the low-level controller is trained end-to-end to generate action commands for local navigation, and the high-level path planner computes the path to the goal for the low-level controller to execute. Kato et al. [48] proposed a navigation system that performs the global navigation based on topological maps and the local navigation based on DRL. In this work, we propose a navigation system that consists of a global planner and a local planner which are both based on learning-based approaches.

## 3. Materials and Models

The proposed navigation system consists of a global planner and a local planner which are based on two different learning methods: end-to-end learning and reinforcement learning. We will briefly introduce them in this section.

### 3.1. Goal-Directed End-to-End Learning

The objective of end-to-end learning is to train a controller to mimic an expert’s behaviors. It is based on the idea that if an expert is able to solve a given task successfully and a controller that mimics the expert’s behaviors will also perform well in this task. For a controller that interacts with the environment at discrete time steps, at each time step *t*, the controller receives an observation $o_{t}$ and takes an action $a_{t}$. Given a set of observation-action pairs {$\langle o_{t}$, $a_{t}\rangle$} generated by an expert, the standard end-to-end learning aims to find a function approximator that maps the observations to the actions. This is a purely supervised learning problem, where the parameters θ of the function approximator F(o;θ) are optimized according to:

(1) $$\underset{\theta }{minimize}\sum_{i}\ell \left(F\left(o_{i};\theta \right),a_{i}\right)$$

This equation is based on the assumption that the expert’s actions are fully explained by the observations, which may hold in simple tasks like lane following and obstacle avoidance but usually breaks down in more complex scenarios. Consider a robot reaching an intersection, the robot’s subsequent actions are not just explained by the local observation, but are additionally affected by the robot’s intended destination. The same observations could lead to different actions. To address this, we explicitly incorporate the intention/goal together with the observation to explain the action. In our goal-directed navigation task, the goal represents the target room the robot is required to enter, which is represented by a vector *g*. At training time, the goal representation is provided by the expert and the controller is trained to adjust its prediction of the action based on this information. The objective of the goal-directed end-to-end learning is

(2) $$\underset{\theta }{minimize}\sum_{i}\ell \left(F\left(o_{i},g_{i};\theta \right),a_{i}\right)$$

In contrast to Equation (1), the predicted action is based on not just the observation but also the goal.

### 3.2. Implementation of the Goal-Directed End-to-End Learning

**Figure 1:**
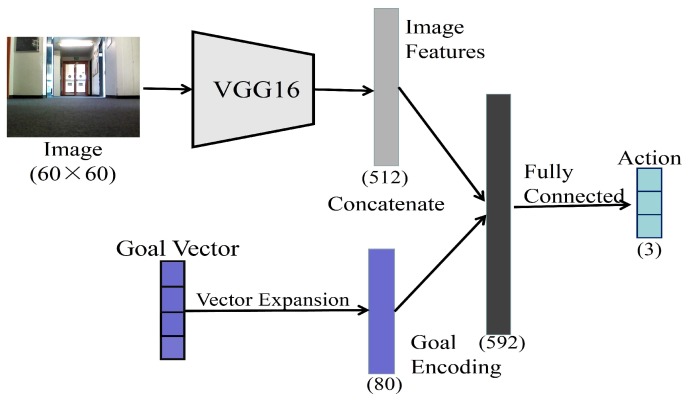
Network architecture for the goal-directed end-to-end learning. The image and the goal representation are concatenated to generate the action.

Figure 1 shows the sketch of the network architecture for the goal-directed end-to-end learning. The network takes as input a camera image of size 60 × 60. It extracts from the image a feature vector of length 512, using pre-trained VGG16 without the last fully connected layer [49]. In addition, the goal representation is encoded by a one-hot vector of length 4, which represents the 4 different room types that are potential to be the goal. This is in accord with the number of semantic categories in the experimental area. The vector is then expanded into a vector of length 80 in order to balance the influence of the visual and the goal representation vectors. The visual features and the goal representation vectors are concatenated together as a joint vector, which is then processed by a fully connected network, which uses RELU nonlinearities and 50% dropout, to generate the actions. The action output is encoded by a 3-dimensional vector that represents turning left, moving forward and turning right. Previous works [15,24] have shown that robots with these three discrete actions are able to navigate through a large environment effectively.

### 3.3. Reinforcement Learning for Local Object Avoidance

In a given environment, the goal-directed end-to-end learning based global planner is able to generate actions towards the goal. However, it is trained in a purely supervised way, where it only imitates the expert behaviors in a relatively static situation. It can not deal with potential changes in the environment, for example, regional changes caused by newly placed objects. To solve this problem, the local planner is built to complement the robot’s ability in dealing with situations. The local planner is responsible for avoiding objects in the local area and is trained based on RL.

### 3.4. Deep Q-Learning

We adopt the deep Q-learning to train the local planner. We formalize this task as a Markov Decision Process (MDP), where the robot interacts with the environment through a sequence of observations, actions and reward signals. For each time step t, the robot perceives a state $s_{t}$ and needs to select a possible action $a_{t}$ according to a policy π, where the π is the probability of selecting an action *a* to be performed for a given state *s*. Once the action has been executed, a positive or a negative value, which may not be delivered immediately, will be provided as a reward $r_{t}$ for the robot by the environment together with the next state $s_{t+1}$. During learning, the robot’s aim is to find a policy that collects the highest reward possible over the long run. Given a policy π, the action-value (Q-value) of a state-action pair (s,a), which indicates the expected total discounted reward when executing actions following policy π from state *s*, is defined as follows:

(3) $$Q^{\pi }\left(s,a\right)=E\left[\sum_{t=0}^{\infty }\gamma^{t}r_{t}|s_{0}=s,a_{0}=a,\pi \right]$$

where the expectation is with respect to the transition distribution under policy π and $r_{t}$ is the reward for action $a=a_{t}$ under the policy π in the state $s=s_{t}$. γ is the discount rate determining future action’s influence (0<γ<1). The Q-value function can be computed using the Bellman equation

(4) $$Q^{\pi }\left(s_{t},a_{t}\right)=r_{t}+\gamma E\left[Q^{\pi },\left(s_{t+1},a_{t+1},\pi \right)\right]$$

The optimal $\pi^{*}$ corresponds to taking the best action in any state *s* and the optimal Q-value function $Q^{*}$ can be obtained as follows:

(5) $$Q^{*}\left(s,a\right)=r_{t}+\gamma \max_{a^{\prime }}E\left[Q^{*}\left(s_{t+1},a^{\prime }\right)\right]$$

where $a^{\prime }$ represents the possible actions in the future state $s_{t+1}$.

The basic idea behind many RL algorithms is to estimate the Q-value by iteratively updating based on the Bellman equation. Traditional methods usually calculate the Q-value function directly over a large state, which has low efficiency and lacks generality. Recent successes of RL in many applications rely on the technique of combing deep neural network and RL, where neural networks are used to estimate the Q-value function. This is the main idea behind DQN [11]. For a neural network that works as a function approximator for the Q-function, its parameters are updated as follows:

(6) $$\theta_{i+1}=\theta_{i}+\alpha \left(r+\gamma \max_{a^{\prime }}Q\left(s^{\prime },a^{\prime };\theta_{i}\right)-Q\left(s,a;\theta_{i}\right)\right)\nabla_{\theta_{i}}Q\left(s,a;\theta_{i}\right)$$

where $\theta_{i}$ are parameters of the network at iteration *i* and α is the learning rate.

### 3.5. Double Q-Learning

As shown in Equation (5), the objective of the Q-learning is to bring the current value of Q(s,a) to the target value of $Y_{t}^{Q}=r+\gamma \max_{a^{\prime }}Q\left(s^{\prime },a^{\prime }\right)$. During the learning process, the Q-function Q(s,a;θ) that evaluates the future approximated action values is also used to select the action. This can sometimes overestimate the action values, resulting in overoptimistic value estimations and slow learning speed. To solve this problem, Van Hasselt et al. [29] proposed the Double DQN (D-DQN) that uses two sets of weights θ and $\theta^{-}$, where the online network (Q(s,a;θ)) is used to select the action and the target network ($Q(s,a;\theta^{-})$) is used to evaluate the action values. The implementation only requires a minor change to the DQN algorithm. Recall that the target in the DQN is calculated as:

(7) $$Y_{t}^{DQN}=r+\gamma \max_{a^{\prime }}Q\left(s^{\prime },arg\max_{a^{\prime }}Q\left(s^{\prime },a^{\prime },\theta \right),\theta \right)$$

The target in D-DQN can be written as follows:

(8) $$Y_{t}^{DDQN}=r+\gamma \max_{a^{\prime }}Q\left(s^{\prime },arg\max_{a^{\prime }}Q\left(s^{\prime },a^{\prime },\theta \right),\theta^{-}\right)$$

where θ is a set of parameters for the online network and $\theta^{-}$ is a another set of parameters for the target network. During learning, θ are updated at every training step while $\theta^{-}$ are fixed over a short period and then copied from the weights θ. D-DQN has been found to learn better policies than DQN on Atari games [29].

### 3.6. Dueling Q-Learning

The Q-value Q(s,a) corresponds to how good it is to take a certain action given a certain state, which implicitly contains two elements: the value of being at the state and the advantage of taking the action at that state. For a state with multiple action choices, DQN usually aims to estimate the Q-value of each state-action pair. However, sometimes it is unnecessary to calculate the value of each action, considering that for states where their actions do not affect the environment in any relevant way. With the aim to explicitly separate the state value and the action advantage, Wang et al. [30] proposed the dueling network architecture. In this architecture, two streams of networks are used to separately estimate the state value function V (s) and the associated advantage function A(s, a), which are then combined together to estimate the action-value function Q(s; a). The Q-value can be constructed as the sum of V(s) and A(s,a)

(9) Q(s,a)=A(s,a)+V(s)

It has been demonstrated that, compared with DQN and D-DQN, the dueling DQN can lead to faster learning speed and better performance in a number of tasks [30].

### 3.7. Implementation of D3QN

RL requires huge amounts of data and time for obtaining appropriate behaviors. For the avoidance behavior learning, actions that collide with obstacles need to be iterated, which is not possible for a robot in the real world. A feasible solution is to implement the training in a simulator and then transfer the learning results to the real robot. However, this is a challenging task considering the huge difference between the structural simulation environment and the highly complicated real-world environment, especially for vision-based learning. In this work, the local planner is trained based on the laser scan data. Compared with visual images, laser scans are relatively low-dimensional and the difference between the simulation and the real world is smaller. It is possible to enable an easier transfer from simulation to reality.

**Figure 2:**
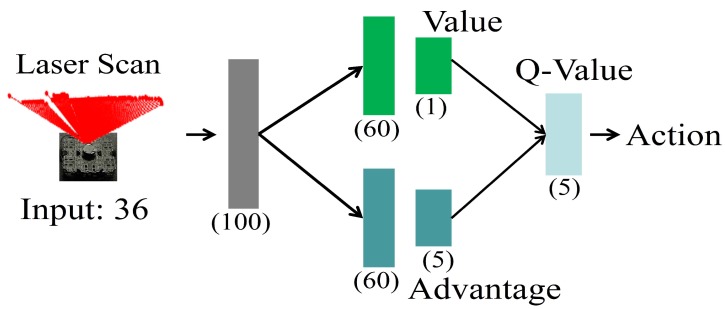
Deep double Q network (D3QN) network structure. Input is a 36-dimensional vector representing the laser range finderings sampled from the raw laser scan between $-180^{\circ }$ and $180^{\circ }$ at 5 degree resolution. The output is a 5-dimensional vector representing the Q-values of the state-action pairs.

In this work, we adopt the D3QN model [32] that combines the double and dueling techniques to train the local planner to perform obstacle avoidance. The architecture is shown in Figure 2. and corresponding implementation details of each layer are summarized in Table 1. The input is a 36-dimensional vector consisting of 36 laser range findings which are sampled from the raw laser range findings between −$180^{\circ }$ and $180^{\circ }$ in a fixed angle distribution of 5 degrees. Each representative range in the input is normalized to [0.0, 1.0]. After the input layer, a fully connected layer of 100 nodes is shared by the value network and the advantage network which both consist of 2 fully connected layers and calculate the value and advantage, respectively. The value network has 1 output and the advantage network has 5 outputs referring to the number of valid actions. The outputs of these two networks are finally combined to compute the Q-values of the state-action pairs.

### 3.8. Switching Between Two Different Strategies

**Figure 3:**
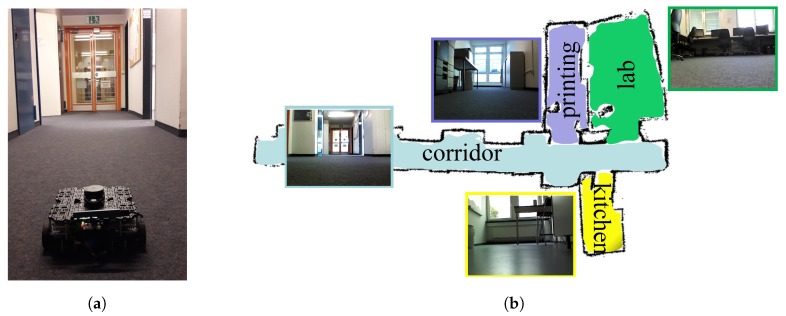
Robot setup and experimental environment. (**a**) The robot (TurtleBot3 Waffle) in the testing environment. The robot is equipped with a $360^{\circ }$ laser distance sensor (LDS) and an Intel RealSense depth camera. (**b**) A semantic representation of the experiment environment, which is a part of our lab. The colors encode the semantic categories of different places, which include four different room types: a corridor (light blue), a kitchen (yellow), a printing room (purple), and a student lab (green).

The local planner works complementarily to the global planner to deal with the changes in the environment. This requires the robot to know the appropriate timing of switching between these two planners. In this work, we use the distance information as an indicator to switch between the global and local planners. Specifically, when the measurements of the robot’s laser range findings in the range of [−$45^{\circ }$, $45^{\circ }$] are smaller than a certain threshold, which means that the robot is approaching an object that is in front of it, the local planner will start working. Since the spaces in the testing environment are quite open during training the global planner, as shown in Figure 3b, and the global planner usually keeps the robot’s moving direction towards the open space, it means that the newly place objects in these areas can be easily detected using the proposed method. In terms of when to stop the local planner, we let the local planner stop automatically after executing a fixed number of steps. The choice of this number of steps depends on the size of the objects and the environment. In our real-world experiments, we let the local planner start working when the robot detects objects within 70 cm in front of it and each time the local planner works 50 steps.

## 4. Experimental Results

### 4.1. Experimental Setup

We demonstrated and evaluated the performance of our proposed navigation system on a real robot, the Turtlebot3 WAFFLE as shown in Figure 3a, in our lab. The robot is equipped with a 360 Laser Distance Sensor (LDS) for acquiring laser scan data and an Intel RealSense depth camera for capturing RGB images. The 360 LDS is a 2D laser scanner capable of sensing $360^{\circ }$ and its maximum measurement distance is 3.5 m. The Intel RealSense camera is set to record images at 640×480 resolution. The open source framework Robot Operating System (ROS) [50] is used to integrate the test system and all the codes are running on a low-performance laptop with an Intel Core i3-4150 CPU. The testing area is part of our lab, on the second floor of a building, and contains 4 different room types including a corridor, a kitchen, a printing room, and a student lab, as shown in Figure 3b. During experiments, the robot started from the corridor and needed to move into one of the four room types according to the given goal, where the robot needed to make the correct turning at certain intersections and to deal with the potential changes in the environment.

### 4.2. Global Planner for Navigating Towards the Goal

#### 4.2.1. Data Preparation and Training

End-to-End learning aims at mimicking an expert’s behavior, where an important aspect is to learn an expert’s ability to recover from mistakes. This requires the training data to include not just observations of expert trajectories but also observations of recoveries from mistakes. For this aim, we adopt the idea of using the three-camera setup in Bojarski’s work [15], where the vehicle is instrumented to record from three cameras simultaneously: One shifted to the left, one facing forward and one shifted to the right and recordings from these three cameras are used to simulate recovery from shifts. However, our WAFFLE robot is equipped with only one camera. For the same effect, when controlling the robot to navigate between different rooms with a joystick, we drove the robot to move along the same trajectories three times. Each time we manually shifted the camera’s direction respectively to the forward, to the left and to the right and recorded the corresponding data for training. While driving the robot, we also recorded the desired room the robot was going to enter. In this work, the robot always started from the same position in the corridor and its goal was to reach the center of the rooms or the end of the corridor in the testing environment, as shown in Figure 3b. For the goal representation, we used a one-hot vector to encode the target room. For example, the corridor is represented by [1,0,0,0], the printing room is represented by [0,1,0,0], the kitchen is represented by [0,0,1,0], and the printing room is represented by [0,0,0,1]. In total, we collected 18,000 images together with corresponding goal representations. The data collection happened several times at different times on different days, during which the environmental structure and the placement of objects were kept almost the same.

During training, the images were resized from 640×480 to 60×60 before being input to the network. For a dataset with a limited number of samples, data augmentation has shown as a good method to increase the data’s effectiveness, preventing overfitting and enabling better generalization. For this aim, we performed online augmentation during training. Specifically, before being fed into the network, each image had to go through a series of transformations including changes in contrast and brightness, and the addition of Gaussian blur and Gaussian noise. The magnitude of these changes is randomly sampled from a normal distribution. However, geometrical augmentations such as translation or rotation were not applied, since action commands are not invariant to these transformations. Given an input image, the output of the model is a probability distribution over all possible actions. We formalized this as a classification problem. We trained the network by minimizing the cross-entropy loss between the predicted action and the desired action. For training the network, we used the Adam optimizer, which is a variation of stochastic gradient descent but usually allows for faster learning, with mini-batches of size 32 and initial learning rate 0.0001, which was then scaled by a factor of 0.99 per epoch. Considering that the training data is highly biased towards moving forward, every time a mini batch was loaded from the training set, we performed data balancing by randomly selecting only a certain proportion of data from this particular category. This enables the date in the batch with a balanced distribution. Training took a couple of hours on a workstation equipped with Nvidia GTX 980 GPU.

#### 4.2.2. Results of the Global Planner

**Figure 4:**
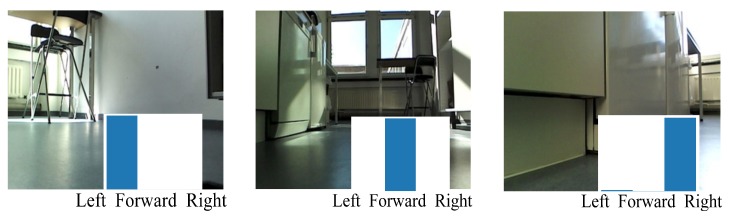
An example of the output of the end-to-end network. The underlying bars demonstrate the action probability predicted by the network.

At test time the trained network was evaluated on the Waffle in real time. It received images from the robot’s camera and goals from the user. Based on this information, the network predicted actions for the robot to execute. The whole process was implemented based on ROS. We first test whether the trained network could keep the robot moving forward and recover from the shift. For this aim, we put the robot in a particular room and limited the robot to move in this particular room by setting its goal vector as the one that represents the current room type. For example, when the robot was moving in the corridor whose goal representation is [1, 0, 0, 0], its goal vector was also set to be [1, 0, 0, 0]. Figure 4 shows three representative frames from a random position in the testing environment. As we can see, the end-to-end network was able to predict the correct actions to keep the robot moving forward.

**Figure 5:**
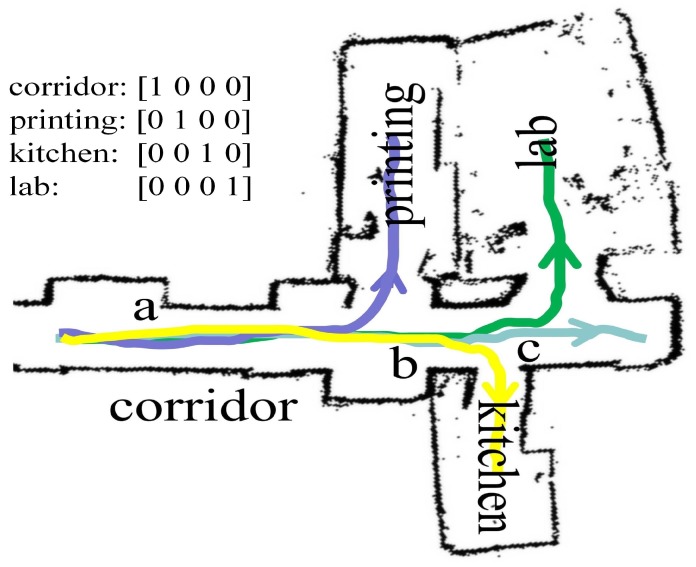
The moving trajectories predicted by the goal-directed end-to-end network towards different goals in the real world. The light blue line represents the trajectory to the corridor end. The yellow line represents the trajectory to the kitchen. The purple line represents the trajectory to the printing room. The green line represents the trajectory to the student lab.

**Figure 6:**
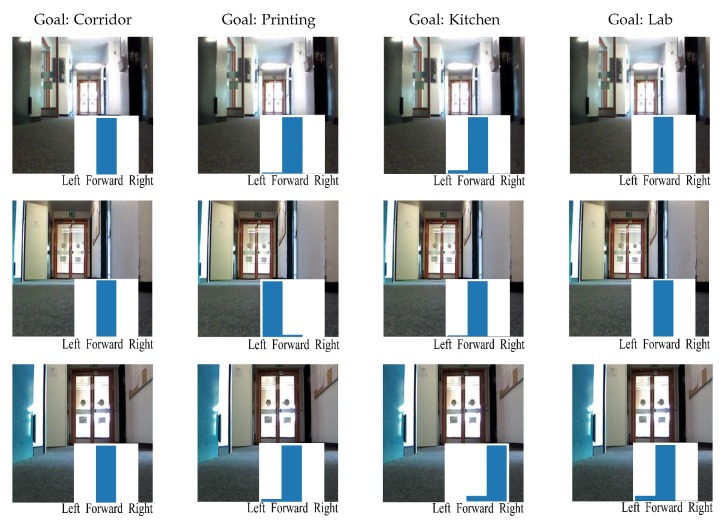
The goal-directed end-to-end network results for three representative positions in the environment. The underlying bars demonstrate the action probability predicted by the network. The rows represent the camera image at three positions sampled from the area a, b, c in Figure 5. The 4 columns represent the 4 different goals: The corridor, the printing room, the kitchen, and the lab.

Figure 5 shows the exemplary trajectories predicted by the goal-directed end-to-end network towards different goals in the testing environment. As we can see, the robot could reach the center of the target room or the end of the corridor successfully. In addition, we present the output of the goal-directed end-to-end network at three representative positions in the environment, which are from the area *a*, *b*, and *c* in Figure 5. As shown in Figure 6, given the same camera image at certain positions, the model could generate different actions according to the goal. This confirms the importance of the goal representation in predicting the actions. Notice that the robot’s action space contains no backward movement. In cases when the robot could not navigate to the target room, we required the robot to move forward. For example, when the robot was in position c, it is not possible for the robot to navigate to the printing room whose goal representation is [0,1,0,0], it would just move forward, as shown in the sub-image of Figure 6 (the third row, the second column).

We compared the performance of the goal-directed end-to-end learning with two baselines that include the standard end-to-end learning and the classic SLAM technique. For the standard end-to-end learning, the training data and procedure were the same as in the goal-directed end-to-end learning, except that the action was predicted based only on the image without the goal representation. For the SLAM navigation, we adopt the ROS navigation package which provides an easy implementation for SLAM. We first drove the robot to explore the testing area to build an occupancy map and then let the robot perform navigation based on the built map. In this work, we performed the laser-based SLAM since it is easier to perform navigation. During the comparison experiments, the robot started from the same position in the corridor and was asked to navigate to the target room in the testing environment based on different approaches. We selected each of the four room types as the target room and conducted ten tests for each one. We defined a test was successful if the robot reached the center of the target room or the end of the corridor. The results and related comparisons are present in Table 2. The goal-directed end-to-end learning clearly outperforms the standard end-to-end learning in navigating towards the desired room and its performance is even comparable to the classic SLAM. Notice that the standard end-to-end learning is based on the RGB camera and SLAM is based on the LDS. Although the SLAM navigation does not need training processes, it requires a metric-precise occupancy map to perform planning. For many vision-based SLAMs, the computational cost of building an occupancy map is huge and the generated map cannot be directly used to support planning and navigation. In contrast, the goal-directed end-to-end learning only requires an abstract topological map during the training process and is able to generate action commands based on the robot’s camera images. During experiments, the standard end-to-end learning usually made arbitrary decisions at intersections and its learning result was highly biased towards going straight. This explained why it had a high success rate in navigating towards the corridor considering that the corridor in the testing environment is straight. For all the failed cases, the robot still demonstrated a good ability to recover from shifts, without colliding into the environment.

### 4.3. Local Planner for Object Avoidance

In order to show the validity of the D3QN-based local planner in learning and performing object avoidance behaviors, we conducted experiments in both the simulation and real-world scenarios. The experiments mainly include training in the simulation and testing in the real world.

#### 4.3.1. Training in Simulation

**Figure 7:**
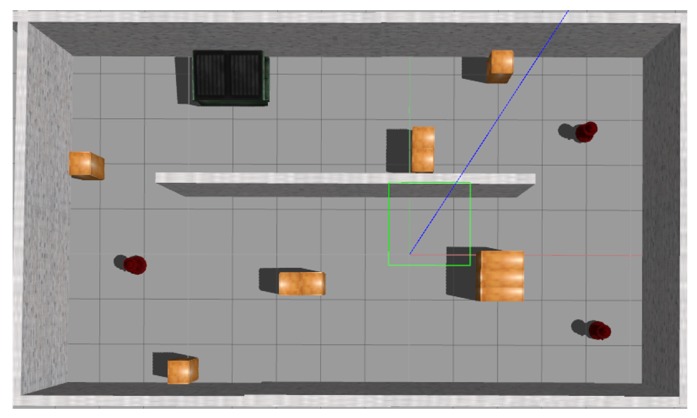
The top view of the simulation environment in Gazebo used for training.

The training procedure of the local planner was implemented in a virtual environment simulated by Gazebo [51]. Figure 7 shows an overview of the simulation environment which contains a number of obstacles of different shapes and sizes. A Turtlebot was rendered as the robot platform and interacted with the environment using its laser sensor. We adopted the D3QN model to train the local planner and related training settings of this learning task are shown as follows:

- Action space: In this navigation task, the robot is instructed to move forward with a constant step length (0.03 m) and the actions are defined to control the robot’s angular velocity in a discretized format. It includes 5 actions: Turning left by $60^{\circ }$, turning left by $30^{\circ }$, moving forward, turning right by $30^{\circ }$, turning right by $60^{\circ }$. In contrast to the global planner that has only 3 action behaviors, the local planner has a more fine-grained action resolution, which is more convenient for local manipulations.
- Observations and Goals: The state of the robot is represented by 36 sampled laser range findings from the raw laser scan. During navigation, the robot’s objective is to learn the action policy that enables the robot to bypass the objects placed in the environment. Since the robot moves with a constant linear velocity, the learning task basically requires the robot to change its angular velocity based on the relative spatial positions between itself and the objects.
- Reward: For each step, the immediate reward is 0.1. If a collision is detected, the episode terminates immediately with an additional punishment of −20. Otherwise, the episode lasts until it reaches the maximum number of steps (200 steps in our experiments) and terminates without punishment. Also, simply rotating on the spot will be punished. The reward function is designed to let the robot move in the environment without colliding into walls and objects as long as possible. The total episode reward is the accumulation of instantaneous rewards over all steps within an episode.

During training, a series of processes from action selection to learning were iterated. In every episode, the robot was initialized at a random position with a random orientation in the simulator, which was guaranteed to be collision-free with objects. The robot navigated through the simulator episodes by episodes, during which the parameters of the neural network were updated based on the interactions with the simulator. To train the network, we used the Adam optimizer with a learning rate of 0.0001. The action selection policy was based on ϵ-greedy with ϵ annealed linearly from 1 to 0.1 over the duration of the training. An experience memory of size 5000 was built to store experiences and mini-batches of 32 were used to randomly retrieve experiences from the experience memory for learning and updating the neural network parameters. During experiments, we found adding noise to the training data could make the trained models transferable better from simulation to reality. For this aim, the laser scans for training in the simulator were corrupted with noise randomly sampled from a normal distribution with parameters mean = 0, std = 0.1.

#### 4.3.2. Results in the Simulation

**Figure 8:**
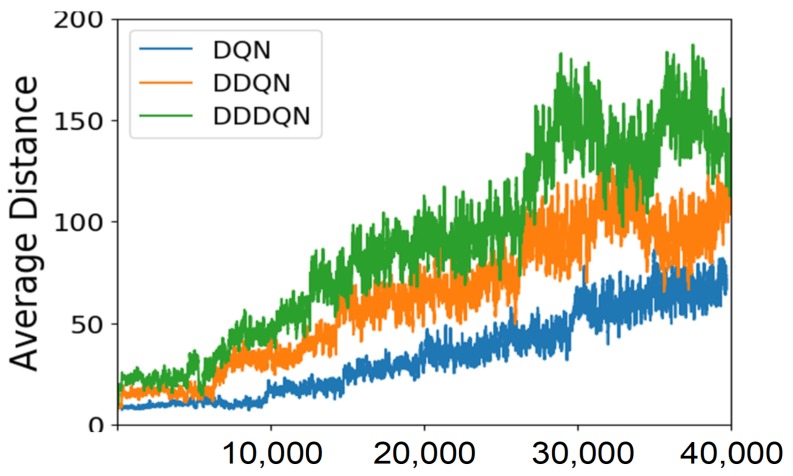
The number of steps performed by the robot without collision in the simulator over learning episodes (smoothed).

Figure 8 presents the learning result in the Gazebo simulator. As we can see, the moving distance of the robot without collision in each episode keeps increasing as the training continues. This means that the robot is learning well on how to avoid objects. In particular, the performances of two baseline systems: DQN and D-DQN, are presented in comparison with the performance of the adopted learning paradigm: D3QN. The learning results show that the D3QN model outperforms the other two in both the learning speed and performance, which demonstrates the advantages of introducing doubling and dueling techniques for learning.

#### 4.3.3. Results in the Real World

**Figure 9:**
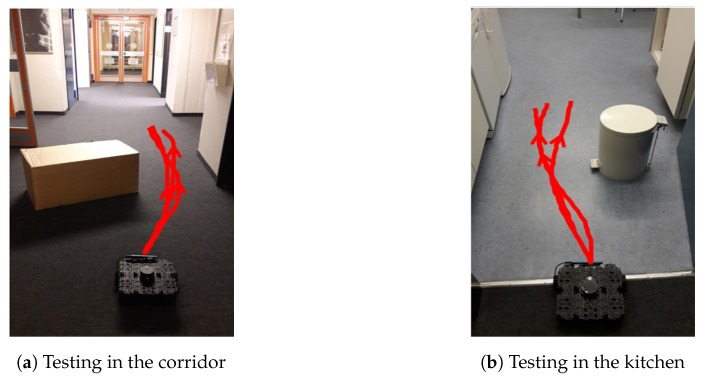
Tests of the local planner in the real world. The red lines represent the robot’s moving trajectories calculated by the local planner. For each scenario, we show three trajectories in three successful tests.

**Figure 10:**
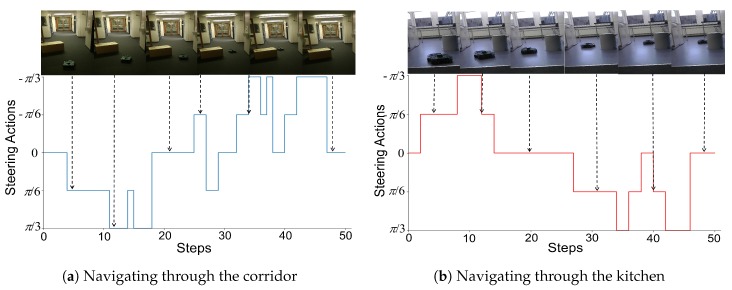
Action choices of the local planner during local navigations. The curve below the image streams shows the steering action selected by the local planner at each step.

After the trained local planner had demonstrated good performance in the simulator, it was loaded on the WAFFLE robot to test its performance in the real world. We conducted real-world experiments in two different scenarios including a corridor and a kitchen. As shown in Figure 9, we put a rectangular box in the corridor (Figure 9a) and a cylindrical trash bin in the kitchen (Figure 9b). These two objects were not seen during training the global planner. In the experiments, the robot’s objective was to bypass the object in front of it while moving forward. Since the local planner only works on a local scale, we only tested its performance in a relatively small area. While the local planner working, the robot moved with a constant linear velocity (0.03 m/s) and we let the local planner work for a fixed number of 50 steps. This number was chosen based on our experimental experience in our testing area. Figure 9 presents several exemplary trajectories calculated by the local planner during the real-world experiments. In addition, Figure 10 presents the steering actions predicted by the local planner from one of the experiments when the robot was passing these areas. As we can see, the robot can navigate smoothly through these areas. Although the predicted steering actions can be a little bit unstable in the corridor (Figure 9a), the robot is still able to navigate through the narrow space between the box and the wall successfully. Experimental results validate that the D3QN based local planner which was trained in the simulation environment is able to perform object avoidance in the real world.

In addition, we compared the performance of the global planner, the local planner and the classic SLAM in dealing with these newly placed objects. In the same testing scenarios, we loaded different models on the WAFFLE and let it navigate through these areas by executing actions generated by them. Experiments in each testing area were repeated 10 times. Table 3 shows the results of the experiments and the comparison between different approaches. The global planner has a very low success rate in the experiments. For example, the global planner totally failed in passing the object in the corridor, considering the only traversable area is the narrow space between the box and the wall. In contrast, the local planner is able to deal with these situations efficiently. Its performance is almost comparable to the standard SLAM and we believe that its performance can be better with more training efforts. Importantly, SLAM requires a prebuilt map to perform planning, while the local planner is free from this requirement. This means that the local planner has lots of flexibility in performing object avoidance behaviors.

### 4.4. Combining the Global and Local Planners

**Figure 11:**
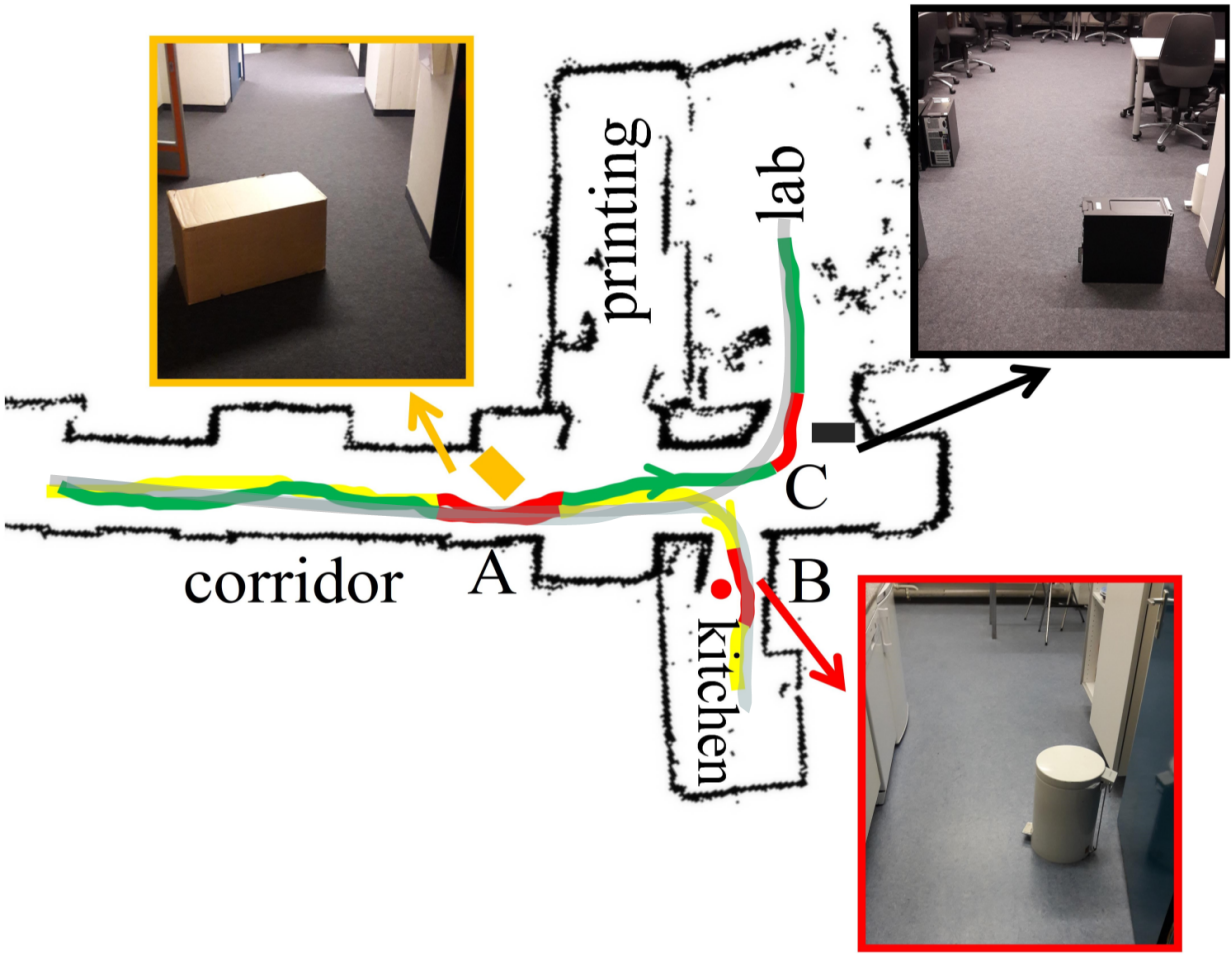
Experiment results of combing the global and local planners. The inset images show the scenes of several newly placed objects in the environment. The green and yellow lines represent the trajectories calculated by the global planner, where the goal of the green one is the student lab and the goal of the yellow one is the kitchen. The red lines represent the trajectories calculated by the local planner which works temporarily to deal with changes in the environment. The light black lines represent the trajectories calculated by the SLAM package.

Finally, we conducted real-world experiments to evaluate the performance of the proposed navigation system that combines the global and local planning in goal-directed navigations. The testing area is the same as the one shown in Figure 3b, except that several objects are placed in the environment. As shown in Figure 11, three objects of different size and shape were placed in area A, B, and C, respectively. Previous experiments have already shown that the global planner could not work well in these scenarios. Figure 11 presents the trajectories calculated by the local and global planner together towards different goals. For comparison, we also present the trajectory calculated by the SLAM package which requires a pre-built occupancy map to perform path planning. It shows that by combining the global and local planners the robot can successfully navigate to the goal in environments with newly placed objects, though the trajectories are not as smooth as the ones calculated by SLAM, where these two planners work alternatively to generate actions for the robot.

## 5. Conclusions

In this paper, we proposed a goal-directed navigation system consisting of a global planner and a local planner, which were both built based on learning-based approaches. The global planner was trained based on the goal-directed end-to-end learning to imitate an expert’s navigation between different areas, where both the goal and local observation were incorporated in generating actions. This enabled the robot to generate actions conditional on the goal. While the global planner failed to deal with changes in the environment, the local planner based on deep RL was built to compensate it. The local planner was specialized in avoiding objects in local areas and worked temporarily in situations that the global planner would easily fail. The local planner was trained in the simulation environment and then transferred to the real world. Experimental results showed that the proposed navigation system could navigate the robot to the goal position effectively in an environment with regional changes.

There are several improvements can be made to the current system. First, the laser-based local planner can be replaced by an image-based one. This will lead to a purely vision-based navigation system. Also, a local planner with continuous action spaces is more natural and flexible in dealing with local manipulations. Therefore, learning algorithms for continuous action spaces should be considered, such as Deep Deterministic Policy Gradient (DDPG) [28] and Asynchronous Advantage Actor-Critic (A3C) [31]. In addition, the local planner can be extended to deal with dynamic objects. Currently, the switching between the global and local strategies is over-simplified in order to facilitate real-world implementation. We believe that a more natural switching mechanism can be developed by utilizing the visual system which plays a crucial role in deciding novel or familiar scenarios.

Our goal-direction navigation system takes advantage of the high-level semantic behind a semantic map. The emerged semantic involves human concepts, such as the types of rooms and their spatial arrangement, which is crucial for the robot to be able to communicate with humans. One interesting direction for future work is to exploit the high-level semantic to contribute to the navigation and human-robot interaction. For example, we can integrate the proposed navigation system with a natural language model so that the robot can perform tasks specified by natural language instructions, like “go to the room with an oven”, where the target room kitchen is inferred using its semantic knowledge.

References

## Figures and Tables

**Table 1:** Implementation details of the deep double Q network (D3QN) for obstacle avoidance.

Layer Name	Layer Type	Number of Neurons	Activation Type
Input	Dense	36	–
Shared FC	Dense	100	ReLU
FC1 for value	Dense	60	ReLU
FC1 for advantage	Dense	60	ReLU
FC2 for value	Dense	1	Linear
FC2 for advantage	Dense	5	Linear
Output	Dense	5	–

**Table 2:** Comparison of the standard end-to-end learning (Standard), the goal-directed end-to-end learning (Goal-directed), and SLAM in navigating towards desired rooms.

	Success Times/Attempts in Navigating to				Success	Navigation Requirement		
Model	Corridor	Kitchen	Printing	Lab	Rate	Sensor	Training	Map
Standard	7/10	2/10	0/10	1/10	10%	Camera	Yes	No
Goal-directed	10/10	8/10	7/10	8/10	82%	Camera	Yes	Topological
SLAM	10/10	10/10	10/10	10/10	100%	LDS	No	Occupancy

**Table 3:** Comparison of the global planner, the local planner, and SLAM in the local navigation.

	Success Rate	Success Rate	Success	Navigation Requirement		
Planner	in Experiment (a)	in Experiment (b)	Rate	Sensor	Training	Map
The Global Planner	0/10	2/10	10%	Camera	Yes	Topological
The Local Planner	8/10	9/10	85%	LDS	Yes	No
SLAM	10/10	10/10	100%	LDS	No	Occupancy
